# Restriction Site Tiling Analysis: accurate discovery and quantitative genotyping of genome-wide polymorphisms using nucleotide arrays

**DOI:** 10.1186/gb-2010-11-4-r44

**Published:** 2010-04-19

**Authors:** Melissa H Pespeni, Thomas A Oliver, Mollie K Manier, Stephen R Palumbi

**Affiliations:** 1 Department of Biology, Stanford University, Hopkins Marine Station, Oceanview Blvd Pacific Grove, CA 93950, USA; 2 Current address: Department of Biology, 107 Life Sciences Complex, Syracuse University, Syracuse, NY 13244, USA

## Abstract

High-throughput genotype data can be used to identify genes important for local adaptation in wild populations, phenotypes in lab stocks, or disease-related traits in human medicine. Here we advance microarray-based genotyping for population genomics with Restriction Site Tiling Analysis. The approach simultaneously discovers polymorphisms and provides quantitative genotype data at 10,000s of loci. It is highly accurate and free from ascertainment bias. We apply the approach to uncover genomic differentiation in the purple sea urchin.

## Abstract 2

A method for the simultaneous identification of polymorphic loci and the quantitative genotyping  of thousands of loci in individuals is presented.

## Background

Uncovering the genetic underpinnings of adaptive evolution is key to understanding the evolutionary processes that generate biodiversity [1]. The combined use of genome scans and population genetic analyses has been applied in both model and non-model organisms to discover and document the role of specific genes in adaptive evolution [2-6]. Surveys of hundreds to thousands of genome-wide markers identified from SNP databases, microarray-based SNP survey methods, or sequences have been applied in humans, yeast, dogs, the malaria parasite *Plasmodium falciparum*, *Drosophila*, and *Arabidopsis *[7-14]. Based on massive sequencing efforts to identify polymorphisms, these approaches have led to insightful evaluation of genetic adaptation. However, these data sets can be complicated by ascertainment bias [15,16] and have historically required a large investment in SNP development.

Approaches to non-model organisms have also resulted in powerful tools to characterize the imprint of selection across the genome at smaller numbers of loci. Tens to hundreds of anonymous genome-wide markers, such as amplified fragment length polymorphisms or microsatellites, have shown genetic patterns correlated to environmental conditions, indicating local adaptation in organisms, including periwinkle snails, lake whitefish, Atlantic salmon, common frogs, and beech trees [17-21]. These methods require little prior marker or sequence information. However, they are limited by the number of loci that can be examined (usually hundreds) and the focus on anonymous loci limits identification of functionally relevant genes [22].

Genome-wide scans of genetic diversity at tens of thousands of loci have become more accessible for non-model study systems with the development of microarray-based polymorphism detection approaches and as the synthesis of species-specific cDNA and high-density oligonucleotide arrays has become more affordable [23]. Specifically, array platforms have been used to detect single feature polymorphisms (SFPs) and restriction-site-associated DNA (RAD) markers by hybridization to species-specific arrays [24-26]. In these methods, a polymorphism is detected as a binding signal difference between individuals or pooled population samples hybridized to arrays. In the SFP approach, labeled genomic DNA from different samples is separately hybridized to high-density arrays of species-specific 25-bp oligonucleotides. In the case of RAD, two individuals are labeled with different fluorescent dyes and co-hybridized to a single array to identify differences. Each approach has advantages: SFP markers are not restricted to restriction cut sites, and RAD markers can be identified using pre-existing cDNA arrays. However, these approaches generate binary data about the presence or absence of a polymorphism at a locus (rather than genotype data of an individual), and RAD requires pairwise competitive hybridization among samples to identify differences. In addition, these approaches have primarily been applied in inbred, genetically tractable study organisms: yeast, *Arabidopsis *strains, *Drosophila *isofemale lines, stickleback lines, zebrafish lines, and *Neurospora *mold [25-31], with the exception of wild caught *Anopheles *mosquitoes [32].

Another potential approach for generating genome-wide polymorphism data in non-model organisms is the combination of next-generation sequencing with targeted SNP genotyping [33-35]. For example, for a species without a sequenced genome, the transcriptomes of multiple individuals could be labeled and pooled ('multiplexed') and sequenced in a single 454 sequencing run [36]. These sequence data can be used to identify common polymorphisms that can then be assayed across more study individuals using a SNP genotyping platform (for example, Illumina's GoldenGate or Infinium platforms or Affymetrix GeneChips). Though this is an attractive approach, there are two major disadvantages. First, only genes expressed in sampled individuals can be compared; genotypes at other genetic loci cannot be assayed, emphasizing an important balance in 454 transcriptome sequencing - breadth of gene coverage across the genome and depth of coverage necessary for polymorphism identification. Second, ascertainment bias would be introduced by surveying only common polymorphisms identified from a subset of individuals. Rare polymorphisms would not be detected in the sequence data or may be excluded as potential sequencing errors. The importance of rare polymorphisms was recently emphasized in two independent studies on human disease. Data from the complete genome sequences of 14 healthy and diseased individuals suggested that diseases, whether rare or common, were caused by rare mutations [37,38]. As a result, an approach that detects even rare substitutions is advantageous.

For population genomics studies, there is a need for higher resolution genome-wide genotype data free from ascertainment bias and a less cumbersome ability to compare numerous individuals across multiple, wild populations. Though future resequencing technologies may allow genetic studies to map traits or search for adaptive genes by whole genome sequence comparisons [23,39], population level studies require comparing numerous individuals at the same loci. The sequencing coverage necessary to repeatedly sample many individuals across the same large set of loci drives resequencing strategies to be less cost-effective than array-based polymorphism discovery and genotyping assays.

Here we present a generally applicable technique, Restriction Site Tiling Analysis (RSTA), which scans for restriction cut site polymorphisms across the genome of an individual using a microarray platform. The technique requires the sequence of a single genome, transcriptome, or large EST library from which to design a species-specific, high-density microarray. The approach allows simultaneous identification of polymorphic loci and the genotyping of individuals as homozygous for a cut site, homozygous for a mutation in a cut site, or heterozygous at thousands of loci. The approach is free from ascertainment bias and does not require competitive hybridization among individuals to identify polymorphisms. These qualities make it well suited for population genomics studies. Genotype data can be used to calculate F_ST _or heterozygosity, or look for patterns of linkage disequilibrium in two or more populations. We first validate the accuracy of the method in detecting polymorphic loci and genotyping individuals. Second, we explore its application for population genomics studies by comparing the genomes of 20 purple sea urchins from two geographically and environmentally distant populations.

We developed this method using the purple sea urchin, *Strongylocentrotus purpuratus *(Stimpson, 1857), as a model system because we are ultimately interested in studying the balance between gene flow and adaptive evolution along environmental gradients. The purple sea urchin lives in intertidal and shallow subtidal habitats from the cold waters of Alaska to the warmer waters of Baja California, Mexico [40]. There is great potential for genetic mixing because larvae may travel far during a 4- to 12-week development phase [41,42]. In accordance with their high dispersal potential, previous studies have found little or no population structure along the coast of the United States [43,44]. In addition, the purple sea urchin is a highly fecund species [42] and has dramatically large population sizes [45]. Theoretically, these characteristics maximize the effects of natural selection and minimize the effects of random genetic drift, making this species a good system in which to study adaptive evolution across the genome. Finally, the purple sea urchin has a published genome sequence [46] and has been the subject of ecological studies for decades [47,48]. However, little is known about the adaptive potential of purple sea urchins despite their broad latitudinal distribution, ecological importance, and their role as a model species in developmental biology.

The purple sea urchin genome is approximately 800 Mb in size, encoding approximately 28,000 genes. There is a similar number of genes and gene structure as seen in the human genome, about 8 exons and 7 introns per gene with each gene spanning on average 8 kb [46]. Exon size is just over 100 nucleotides and intron size is about 750 nucleotides, shorter than introns in the human genome as expected with the smaller genome size. The species is highly polymorphic relative to other species with sequenced genomes. Using thermal DNA reassociation experiments, it was estimated that two individual urchins differ from each other in about 4% of the nucleotide pairs in single-copy DNA [49]. Genome assembly revealed about one SNP per 100 bases and a comparable number of indel polymorphisms [46] when aligning the sequenced DNA from the single inbred diploid individual sea urchin. Such high heterozygosity has impeded a more complete assembly of the genome. In the most recent build of the genome sequence (Spur_v2.1, September 2006), there were 114,222 scaffolds of which 16,057 had multiple contigs with an N50 of 183 kb. Scaffolds are not physically mapped to chromosomes.

## Results

### RSTA hybridization results

**Figure 1:**
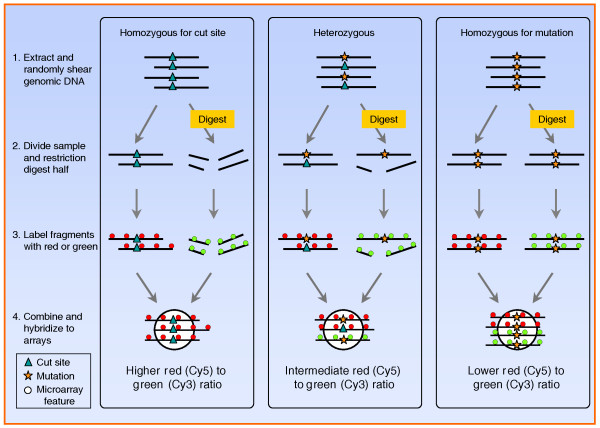
**Restriction site tiling analysis identifies polymorphisms and genotypes individuals by hybridization to a custom microarray**. Fifty base pair tiles (white circles) are designed to be centered on restriction enzyme cut sites. DNA from an individual is extracted and randomly sheared by sonication. The sample is then divided in half: one part is treated with the restriction enzyme and labeled with green fluorescent dye (Cy3), the other part is treated as a control (without restriction enzyme) and labeled with red fluorescent dye (Cy5). The two parts are mixed and hybridized to the array. This DNA processing and hybridization result in different fluorescent signals reflecting the three possible genotypes for a polymorphic locus: when an individual is homozygous for the cut site (blue triangle) the digested DNA is cut and does not hybridize to the tile, resulting in a high red-to-green ratio (log2 Cy5/Cy3, left panel); however, if an individual is homozygous for a mutation in the cut site (yellow star) then the DNA remains intact and hybridizes to the tile, resulting in high green signal intensity or a low red-to-green ratio (right panel). Heterozygous individuals yield an intermediate red-to-green ratio. Polymorphic loci are identified based on the bi- or trimodal distribution of log ratios across sampled individuals. Individuals can be genotyped based on their log ratio.

RSTA is based on differential binding of restriction digested and non-digested DNA from a single individual to a single array with 50-bp tiles designed to be centered on known restriction cut sites (Figure 1). Specifically, for each individual, genomic DNA is randomly sheared by sonication, restriction digested and internally labeled with fluorescent dCTP using random octomers (Cy3, green). Non-digested DNA from the same individual is labeled with a different color (Cy5, red). These genomic preparations from the same individual are then pooled and hybridized under conditions that favor binding of uncut DNA over cut DNA to the array tiles. DNA that matches the known genome sequence is cut by the restriction enzyme, resulting in poor binding to the array tiles, low Cy3 signal intensity, and a high Cy5 to Cy3 ratio. In contrast, DNA with a polymorphic mutation in the cut site remains intact, resulting in a high Cy3 signal intensity, and a more even Cy5 to Cy3 ratio (Figure 1).

We designed several types of tiles in order to confirm that genomic DNA from a diploid organism with a large, complex genome interacted with the array platform as predicted. There were five tile types on the array: restriction cut site centered tiles (n = 50,935), control tiles centered on non-cut sites in single copy genes (n = 10,523), negative control tiles that did not match anywhere in the genome based on BLASTN results (n = 1,036), positive control tiles that matched multi-copy ribosomal DNA (n = 100), and a degradation series to examine the effect of mutational differences between sample DNA and tile sequence on binding efficiency (n = 1,100). We surveyed TaqáI restriction cut sites, though any restriction enzyme or number of enzymes could be used as long as each 50-bp probe is non-overlapping. TaqáI recognizes four base pairs (TCGA) and in doing so is predicted to occur, on average, every 256 bases. The average intermarker distance was 15.7 kb between restriction cut site centered tiles across the 800 Mb genome.

**Figure 2:**
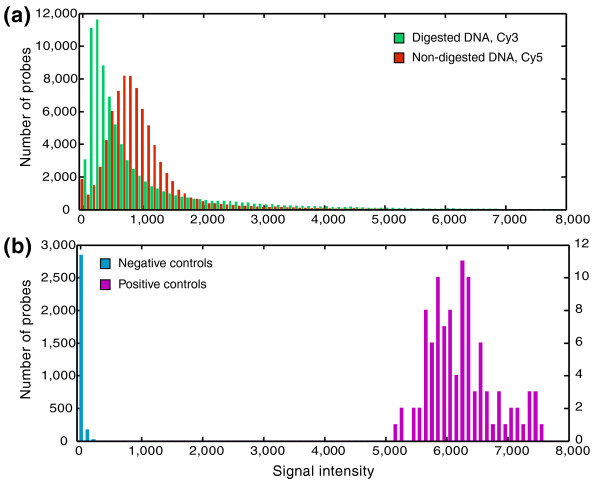
**Frequency histograms of signal intensities for experimental and control tiles**. **(a) **Digested DNA (green, labeled with Cy3) and non-digested DNA (red, Cy5) binding to restriction cut site centered tiles. **(b) **Cy5 signal intensities for negative control tiles (blue, randomly generated tiles that did not match anywhere in the genome according to BLASTN) and positive control tiles (magenta, matching multi-copy ribosomal DNA).

Both experimental and control tiles yielded expected signal intensities (a proxy for binding efficiency). Restriction digestion resulted in a significantly lower distribution of green (Cy3) signal intensities for restriction cut site centered tiles compared to the control red (Cy5) channel (Figure 2a; KS test, *P *< 0.0001). Control non-cut site tiles showed strong Cy3 (digested DNA) signal intensities, indicating no effect of restriction digestion (KS test, *P *< 0.0001). Negative control tiles had very low signal intensities, significantly lower than experimental tiles (Figure 2b; KS test, *P *< 0.0001). Positive control tiles designed to match ribosomal DNA had much greater signal intensity than experimental tiles designed to single-copy loci (Figure 2b; KS test, *P *< 0.0001). We assessed the repeatability of the RSTA approach by performing experimental and technical replicates (that is, independent extraction, processing and hybridization of DNA from a single individual to multiple arrays, and replicate tiles synthesized in triplicate on a single array). These experiments revealed that the signal intensities of corresponding tiles among replicate arrays were highly consistent (R^2 ^= 0.92) and that there was low variance among replicate tiles on a single array (coefficient of variation = 0.08).

### Identification of polymorphic loci

**Figure 3:**
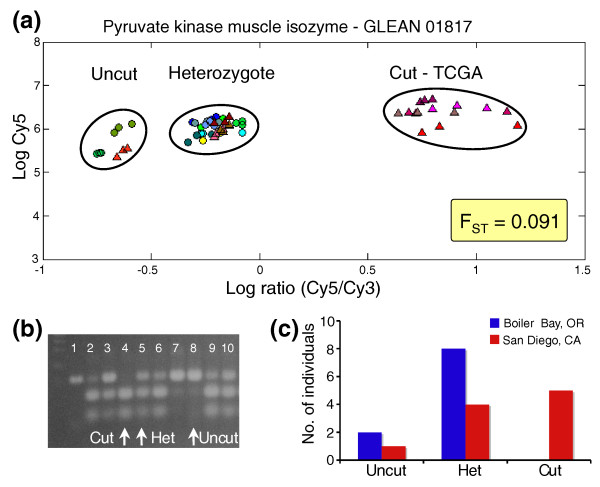
**Polymorphic restriction cut site in pyruvate kinase muscle isozyme across 20 individuals**. **(a) **RSTA array log ratio data separate genotypes of individuals sampled. Cool colored circles represent individuals from Boiler Bay, Oregon; warm colored triangles represent individuals from San Diego, California. The data for each individual are in triplicate. **(b) **Individual genotypes confirmed by restriction digest gels. Lane 1 is an undigested PCR fragment for size reference, while lanes 2 to 10 are treated with the restriction enzyme; lanes 2, 3, 5, 6, 9, and 10 are from heterozygous individuals; lane 4 is from an individual homozygous for the cut site; lanes 7 and 8 are individuals homozygous for a mutation in the cut site. **(c) **Genotype data resulting from RSTA can be used to look for differences across populations.

We compared the genomes of 10 individual purple sea urchins from Boiler Bay, Oregon and 10 individuals from San Diego, California at 50,935 restriction cut sites using 20 RSTA arrays. We genotyped the ten northern sea urchins and the ten southern sea urchins at five known polymorphic restriction cut sites through PCR amplification and restriction digestion and sequencing. We then examined the RSTA array data from 50-bp tiles designed around each of these five loci. We found for each locus that RSTA data across the 20 individuals consisted of three clusters corresponding to the two homozygous and the heterozygous genotypes (Figure 3a). The homozygote clusters were separated by more than 0.7 log ratio units. We used these log ratio characteristics (three clusters and a range greater than 0.7) to identify polymorphic loci among the other 50,930 loci based on their RSTA array data. We used the Bayesian hierarchical clustering algorithm Mclust [50] to determine the number of clusters that best described the log ratio data for the 20 individuals for each locus. These criteria identified 12,431 loci as polymorphic out of the 50,935 loci surveyed (24%). There were 6,859 polymorphisms in coding regions, 2,253 in putative regulatory regions, and 3,319 in intergenic regions. We confirmed individual genotypes for a subset of loci using PCR amplification and sequencing (see below) or restriction digestion gels (Figure 3b). We used the resulting genotype data to look for signals of population differentiation at specific loci (Figure 3c).

### Accuracy of detecting polymorphic loci and genotyping

To determine the accuracy of the RSTA method and to determine the log ratio range for each genotype, we designed primers to amplify and sequence 15 loci, 7 putative polymorphic loci and 8 putative monomorphic loci, across the 20 individuals. We found 99.6% accuracy in genotypes called from RSTA array data (252 correct out of 253 genotypes surveyed). Of the 8 putative monomorphic loci, all were monomorphic; 139 out of 139 (100%) of the genotypes across the 20 individuals were homozygous for the TaqáI cut site (TCGA). Out of the 114 polymorphic genotypes we confirmed with sequence data, 113 (99.1%) matched genotypes called from the RSTA array. From these confirmed genotypes, log ratio data for different genotypes reliably fell into three distinct clusters (less than -0.6 for homozygous uncut, between -0.6 and -0.1 for heterozygotes, and greater than -0.1 for homozygous cut). We used these cutoffs to call individual genotypes among all polymorphic loci from the population data set. These results show that our method of polymorphism identification and genotype calling was highly accurate under these conditions, distinguishing monomorphic and polymorphic loci and correctly calling genotypes of polymorphic loci.

We were also able to detect insertion-deletion polymorphisms (indels) in the RSTA array data. Indels affected the Cy5 (non-digested) signal such that alleles with a deletion had a low binding signal (signal intensity <50), in the same range as background and negative control tiles. Alleles that matched the published genome sequence had a normal binding signal (signal intensity >150, depending on tile sequence). To identify loci with indel polymorphisms, we used these signal intensity cutoffs and the presence of two or three clusters in the Cy5 signal intensity data. We found that 3% of loci in coding regions had indel polymorphisms. We sequence-confirmed one particularly interesting locus, a mannose receptor, and found that RSTA array data matched sequence data in all cases. The sequence data revealed a 3-bp deletion in seven of seven predicted deletions while five out of five sequences matched the tile sequence as predicted. Genes with indels could be top candidates for further study as they likely result in an amino acid sequence change, possibly affecting protein function.

We found that approximately 24% of surveyed restriction cut sites contained a mutation among the 20 individuals surveyed, which equates to about one polymorphism per approximately 200 bp of the purple sea urchin genome. This is less than expected based on the genome assembly, which found at least one SNP every approximately 100 bp and an equal proportion of indels. Due to the high degree of genetic diversity in this species, it is likely that a large proportion of polymorphisms among the 20 individuals sampled went undetected. In highly polymorphic genomic regions, the sampled DNA will not bind to the microarray tile and polymorphisms cannot be detected in the surveyed cut site. This is supported by the observation that we had a significantly greater fraction of tiles with poor binding signal in non-coding regions (7.8%) where higher rates of polymorphism were expected than in coding regions (4.3%, chi-square = 5049.6, *P *< 0.0001). To determine the effect on hybridization of mutational differences between sample DNA and microarray tiles designed from the published genome sequence, we designed tiles that were a perfect match to one place in the genome, then randomly mutated 1 to 10 bases, resulting in a series of 11 tiles per perfect match tile. We did this for 100 perfect match tiles, resulting in a degradation series data set of 1,100 tiles. We found that there was an 80% reduction in signal intensity with four mutational differences in the 50-bp tiles, resulting in near background signal intensity range. These data suggest that 8% sequence difference between a DNA sample and microarray tile results in near complete hybridization loss.

### Population patterns of polymorphic loci

For the 12,431 polymorphic loci, we constructed a genotype matrix for the 20 individuals. We used this matrix to calculate heterozygosity and F_ST_. We found that San Diego individuals had a significantly higher mean heterozygosity (0.2427) than Oregon individuals (0.2258; KS test, *P *= 1.38 × 10^-7^), supporting the hypothesis of higher gene flow (larval dispersal) from the north to the south along the US West coast [51]. As expected, we found a higher frequency of the uncut homozygous genotype (different from the published genome sequence, where the individual sequenced was from southern California) in Oregon individuals (0.1035) than San Diego individuals (0.0869; KS test, *P *= 5.014 × 10^-11^). We used the genotype matrix to calculate F_ST _for each locus as F_ST _= (H_T _- H_S_)/H_T_, using allele frequencies to estimate heterozygosity, where H_T _is the total heterozygosity across populations and H_S _is the mean of heterozygosity within populations [52]. The genome-wide mean F_ST _was 0.0029 among populations, with single locus F_ST _values ranging from 0 to 0.5.

Genome-wide population patterns revealed that all loci were in Hardy-Weinberg equilibrium after multiple test correction. Among the top 100 highest F_ST _coding loci and the top 100 highest F_ST _loci overall, we found no linkage disequilibrium among any locus pairs after multiple test correction (using Genepop [53]). We looked for patterns of linkage in 687 paired loci in coding regions and corresponding upstream regions of the same genes. We found a highly significant correlation between the F_ST _values of the paired loci (correlation coefficient = 0.3288, *P *< 0.0001). These data suggest that similar forces are acting on genetic differentiation in coding and upstream regions, either because of linkage across the two tile sites (2 to 10 kb apart) or the joint action of selection.

### Genetic differentiation along the species range

**Figure 4:**
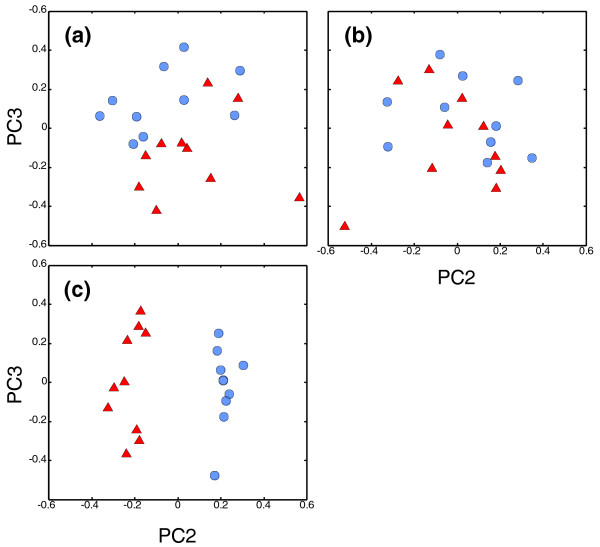
**Principal Components Analysis using RSTA array log ratio data show a signal of population differentiation in a high gene flow species**. Symbols represent individuals from Oregon (blue circles) and San Diego (red triangles). **(a) **All polymorphic coding loci, 6,859; **(b) **polymorphic coding loci excluding top F_ST _loci, 6,555; and **(c) **top F_ST _polymorphic coding loci, 304. Patterns were similar for other tiles in non-coding regions.

We applied Principal Components Analysis (PCA) to determine if there was a signal of population differentiation in the array data set. Analyzing the log ratio data of all polymorphic loci, we found that principal components two and three spatially separated Oregon and San Diego populations (Figure 4a). By removing loci in the tail of the F_ST _distribution (F_ST _>0.1, defined by the mean F_ST _plus two times the standard deviation, approximately the top 4%), we found that the spatial split between populations was lost (Figure 4b). These results suggest that >95% of the purple sea urchin genome has no signal of population differentiation, in accord with previously published descriptions of a few loci [43,44]. As expected, the high F_ST _loci (top 4%) show a strong separation of Oregon and San Diego individuals along PC2 (Figure 4c; see Additional file 1 for a list of the top 100 loci and the corresponding gene annotations).

**Figure 5:**
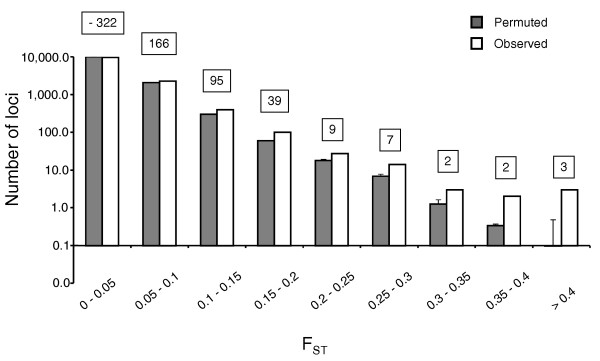
**Genome-wide distribution of F_ST _values**. Open bars show the observed distribution for 12,431 polymorphisms. Solid bars show the mean of 10,000 random permutations. Error bars represent standard deviation for permuted distributions. Numbers in boxes show excess number of loci observed over mean permuted.

Overall F_ST _was low: 0.0029. To test the significance of this value, we randomly shuffled the alleles from all 20 individuals and recalculated F_ST _over 10,000 permutations for each polymorphic locus. We compared the observed genome-wide F_ST _distribution to the permuted distributions to determine if the observed F_ST_s were higher than would be predicted under panmixia. The observed distribution was significantly broader than 9,991 (99.91%) of the permuted distributions (KS test, *P *< 0.0001; Figure 5). The observed mean was higher than the permuted mean (observed: 0.0029 > permuted: 0.0026) over all the 10,000 simulations. The mean and median of the observed distribution was higher than 100% of the simulated distributions. These results show that the observed data consistently had a higher F_ST _than expected under panmixia. Moreover, the observed distribution always had more loci with F_ST _>0.2 than seen in the permuted distributions. The higher levels of F_ST _in the observed data set suggest that there is low but significant genetic differentiation between populations. Such differentiation could be due to low gene flow among populations, selection at some loci, or both.

Detecting loci under selection depends on evaluating the distribution of F_ST_s among loci compared to that expected under neutrality [3]. We searched for loci that showed significantly high F_ST _values using the procedure of Beaumont and Nichols as implemented in LOSITAN [54]. Three significant loci were identified by this analysis (*P *< 0.000002), along with a fourth marginally significant (*P *< 0.00003). These conclusions are limited by the large number of multiple tests, requiring a strong multiple test correction factor, but the distribution of *P*-values suggests selection acts on more loci than just these three. Seven loci show *P*-values < 0.0001 whereas less than one is expected. Likewise, the number of loci with *P*-values < 0.001 or < 0.01 is higher than expected (22 versus 7, and 93 versus 69, respectively).

A separate procedure, in which selection on loci is estimated from the data and the distribution of selection factors (α) is tested against Bayesian expectation, was suggested by Beaumont and Balding [55] and augmented by Foll and Gaggiotti [56]. This test returns three strongly significant loci (Bayes factor >10) - two of which were detected in the previous analysis. The third significant locus is ranked fourth in the previous test. These values show selection factors (α) of 1.3 to 1.4. Simulations suggest that these values correspond to mild selection coefficients (s) of about 0.02 per generation [56]. In summary, our data suggest selection is acting on a small number of loci, but also suggest that selection occurs at other loci as well. In this high gene flow species, increased sampling at the individual and population levels using RSTA or other more targeted approaches would be needed to test robustly for selection across the genome.

The top five genes in which loci were identified as outliers were mannose receptor C1, transcription factor 25, cubilin, a chromatin assembly factor (retinoblastoma binding protein 4 (RBBP4)), and a Golgi autoantigen. Mannose receptors bind to foreign cells and target them for destruction by the immune system [57]. Polymorphisms in mannose-binding proteins in humans are associated with infection frequency [58], but no data exist yet on the role of sea urchin polymorphisms. Transcription factor 25 (TCF25) and the chromatin assembly factor (RBBP4) both negatively regulate transcription. Cubilin is a multi-ligand endocytic receptor important for the endocytosis of proteins, nutrients and vitamins, and is massively expressed in the yolk sac during development [59]. The Golgi autoantigen (Golgin subfamily A member 3 (GOLGA3)) is an autoimmune antigen associated with the Golgi complex and has been shown to be important for successful spermatogenesis [60]. These genes suggest important roles for immunity, transcriptional regulation, and reproduction and development. These processes have previously been shown to be targets of natural selection in other systems [61-63].

Several other particularly interesting genes were among the highest F_ST _loci (Additional file 1) as potential targets of natural selection. These include a toll-like receptor (Tlr2.1), cytochrome P450, receptor for egg jelly 7, and a GABA-receptor, among others. Toll-like receptors and cytochrome P450 are environmental response genes that function during bacterial outbreaks [64,65] and environmental stress [66,67]. Receptors of egg jelly are expressed on the apical tip of sperm heads and are critical proteins in gamete recognition [63]. GABA receptors function in some taxa as signals for larval settlement [68], and could play a role in habitat selection during early life. Alternatively, it could play some other role in larval nervous system function.

## Discussion

### Comparison of RSTA to other high-throughput polymorphism discovery methods

RSTA significantly advances other related high-throughput polymorphism discovery and genotyping methods by providing quantitative genotype data for each individual surveyed for each polymorphic locus identified (Table 1). Such data can be used to examine population allele frequencies at tens of thousands of loci, calculate F_ST _or Hardy-Weinberg equilibrium, model neutrality, identify outlier loci, or apply any other downstream population genetic analysis that requires genotype data. We also demonstrate that RSTA is highly accurate in outcrossed populations sampled from the wild, making it useful for species that cannot be crossed in the lab. The application of RSTA for genome-wide surveys of wild populations can generate hypotheses regarding genes important for local adaptation in species that do not have a visible trait that might confer a fitness advantage.

**Table 1:** Comparison of four high-throughput polymorphism detection approaches

Parameter	SFP	RAD tagging	RAD sequencing	RSTA
Marker type	SNPs and indels	Restriction cut site polymorphisms	Sequence data: SNPs next to restriction cut sites	Restriction cut site polymorphisms: distinguishes SNPs and indels
Number of loci surveyed	92,924	19,200 (elements on an enriched RAD-tag microarray designed from stickleback)	26 nucleotides at 41,622 RAD tags	50,935
Number of polymorphisms identified (informative marker rate)	3,806 (4% at a 5% false discovery rate cutoff)	1,990 (10% at a two-fold signal difference cutoff)	Approximately 13,000 (31%)	12,431 (24%)
False discovery rate	3% (117 out of 121 confirmed correct by sequencing)	9% (20 out of 22 confirmed correct by sequencing)	Not reported	<1% (113 out of 114 confirmed correct by sequencing)
Platform	Custom high-density oligonucleotide array (Affymetrix), 25 bp oligo	cDNA or genomic tiling array (in house synthesis)	Illumina sequencing	Custom high-density oligonucleotide array (Agilent), 50 bp oligo
Prior information required	EST, 454 or genome sequence	EST or RAD-tag library for array synthesis	EST or genome sequence to map short sequence reads	EST, 454 or genome sequence
Polymorphism identification	Hybridization signal difference among study individuals	Hybridization signal difference between two study individuals	Custom Perl scripts for sequence alignment	Genotype clusters across all study individuals
Individual genotype data	No	No	No	Yes
Organisms studied	Yeast, *Arabidopsis, Anopheles*, several seed plants^a^	*Drosophila*, stickleback, zebrafish, *Neurospora*	*Neurospora*	Purple sea urchin

Numbers are from studies that describe each method: SFP [26]; RAD tagging [25]; RAD sequencing [50]. ^a^See Gupta *et al*. [23] for review of high-throughput applications in crop plants.

RAD tagging, like RSTA, surveys the genome of a species for restriction cut site polymorphisms using an array platform [25]. The RAD system compares the hybridization signal between two genome preparations that are co-hybridized, and provides a view of the relative degree of restriction digestion in the two genome preparations. Applying the RAD approach in our study system at the level of individual DNAs would have required 190 hybridizations in order to compare all individuals to one another in the way that 20 RSTA hybridizations allowed. In addition, the resulting 190 RAD hybridizations would produce a qualitative ranking of allele content among individuals, but not the precise genotypes at all loci. Applying the SFP [26] approach, however, though this has not been demonstrated, could yield quantitative data because, like RSTA and unlike RAD, there is no PCR amplification step in DNA processing and each individual is hybridized to a single array. PCR amplification can generate differences in allele copy numbers between samples, making detecting differences between samples qualitative rather than quantitative. However, the short oligonucleotide size (25 bp) in the SFP approach could add noise to the data through non-specific binding, particularly in species with large complex genomes, and could yield more subtle differences between genotypes at each polymorphic locus. This would necessitate large sample sizes to improve the signal to noise ratio for quantitative SFP genotype data. RSTA may be better suited for species with large genomes or high heterozygosity and may yield cleaner data for heterozygotes because of the longer oligonucleotides used (50 bp).

RSTA, RAD, and SFP approaches can be applied to 'bulk' DNA pooled from individuals from a single population. This drastically reduces the number of arrays needed but also reduces the data to a qualitative assessment of gene frequency differences between pooled samples because there is not a precise relationship between hybridization signal difference and gene frequency difference. By contrast, the RSTA approach applied at the individual level allows gene frequencies to be precisely quantified among populations and produces multi-locus data sets of high accuracy at the individual and population levels.

RAD tagging has been extended to use next-generation sequencing to identify polymorphisms [30]. RAD sequencing reduces representation of the genome by sequencing adjacent to conserved restriction cut sites. The approach identifies a similar number of markers as RSTA, although it does not provide genotype data. Half of one Illumina run yielded approximately 0.4- to 1-fold coverage across the 96 individuals studied [30]. An estimated 13-fold coverage is necessary for accurate identification of heterozygotes [69], making next-generation sequencing costly for genotype data at this stage.

In applying RSTA, DNA processing and data analysis is simpler than in other approaches. DNA processing proceeds as follows: shear by sonication, restriction digest with chosen enzyme, fluorescently label, then competitively hybridize with control, non-digested DNA from the same individual. Hybridization against control DNA from the same individual and screening for trimodal data across the population data set nicely separates signal from noise in microarray data, likely resulting in the low false discovery rate (<1%). The RSTA approach can also distinguish SNP and indel polymorphisms using the hybridization signal of the control, non-digested DNA.

The major advantage of the RSTA system is that it produces highly accurate genotypes of individuals at many loci simultaneously without ascertainment bias. Other platforms can provide this information for well-defined systems, though there will be ascertainment bias if targeted SNPs are surveyed - for example, the Affymetrix platform used for humans, dogs, or yeast. In addition, there is a high upfront cost for microarrays that require mask development and there is little chance that such gene chips will become available for many species. In the field of population genomics, there is a need for and keen interest in generating genome-wide genotype data for wild populations of a species. RSTA provides such quantitative genome-wide genotype data in a technically and analytically straightforward approach and without an upfront microarray design cost.

### Opportunities for expanded genome-wide population genetics

We present an accurate genome scanning method that allows simultaneous discovery of polymorphisms and genotyping of thousands of loci by surveying for restriction cut site polymorphisms using an affordable, species-specific microarray. The RSTA array approach can be applied to any species with a cDNA library database or 454 transcriptome sequence, for example. A combination of 454 transcriptome sequencing with a breadth of gene coverage and RSTA polymorphism discovery and genotyping could be very fruitful for the discovery of functionally important genes in non-model species. A breadth of gene coverage in transcriptome sequencing could be accomplished by pooling across multiple tissues and life history stages and tissues sampled after treatment with various environmental stimuli. Because 4-base restriction sites occur at random about every 256 bp (for gene regions with equal nucleotide frequencies), 10,000 kb of sequence data (comparable to what was generated for the Glanville fritillary butterfly using 454 sequencing [35]) would provide on the order of 40,000 RSTA tiles. There is also great potential to increase genome-wide coverage by increasing the number of restriction cut sites surveyed. There is no compromise in data quality in assays of sites from multiple restriction enzymes as long as sites are further than 50 bases apart such that tiles are not overlapping (data not shown).

The application of RSTA in species with lower genetic diversity than purple sea urchins could reveal a lower proportion of polymorphic RSTA tiles. However, the high degree of genetic diversity in purple sea urchins (approximately 4% in single copy genes [49]) may have dramatically reduced the proportion of polymorphic RSTA tiles detected in sections of the genome that have multiple substitutions, largely because such areas may not hybridize well. Thus, in species with less genetic diversity, it could be possible to identify an equal or greater proportion of polymorphisms as were observed in this study, depending on the polymorphism rate in the species and the number of individuals sampled in the study.

The absence of ascertainment bias in RSTA is a major advantage in SNP determination compared to targeted SNP genotyping. RSTA also has the ability to identify rare polymorphisms; the Mclust clustering algorithm defines the number of clusters that best describe the data regardless of the number of data points in each cluster. However, RSTA does not identify all polymorphisms in a gene, and there are many SNPs that remain undetected using this method.

In species without a complete genome sequence, noise could be added to the data by failure to exclude probes that match multiple places in the genome. We excluded approximately 19% of probes due to redundancy when RSTA features were compared back to coding regions. This fraction of redundant probes could also be excluded if using a 454 transcriptome sequence that has a good breadth of gene coverage.

Differences in gene frequencies between two sea urchin populations suggest that *S. purpuratus *is mildly differentiated along the US west coast, just as it is along the coast of Baja Mexico [70]. Previous assays of population structure were derived from relatively few mitochondrial DNA, allozyme or microsatellite loci [43,44,71], and reported no population differentiation except for the southern end of the species range [44,70], or between age classes at one locus [71]. In the present study, population structure is indicated by F_ST _values that are higher than expected, from a greater fraction of homozygous uncut genotypes in Oregon than in California, and a higher heterozygosity in the southern end of the species range. In addition, several loci appear more differentiated than expected under neutral evolution, a result that might be due to natural selection on these loci. Selection on single loci has been inferred in other marine species living across environmental gradients with allozymes [72,73] or through outlier F_ST _analyses [74]. Conclusions about selection from our data are preliminary due to the potential impact of mild population structure on the distribution of F_ST _among loci. However, the outlier loci and highest F_ST _loci play roles in biological processes that we would predict to be important for local adaptation in this species: immunity, transcriptional regulation, environmental response, and reproduction and development.

## Conclusions

We have presented a new genome scanning technique that allows the discovery of polymorphic loci and returns quantitative genotype data at tens of thousands of markers. The approach requires genome or transcriptome sequence data from one individual, though is free from ascertainment bias as polymorphisms are discovered without any prior knowledge by screening all individuals studied. Genotype data can be paired with locus position information to map disease-related or adaptive phenotype-related traits to specific genomic regions or paired with coalescent simulations to identify divergent (F_ST_) outlier loci. This approach, and others like it that generate data on genome-wide distributions of polymorphisms, promises to aid in the identification of ecologically relevant genes and traits in both model and non-model organisms. Such high-throughput genotype data will allow a much greater understanding of the role of environmental variation in shaping genetic diversity patterns and help reveal the genetic basis of adaptive evolution in natural populations.

## Materials and methods

### RSTA array design

We designed 50-bp oligonucleotide tiles by screening the published purple sea urchin genome sequence [46] for TaqαI restriction enzyme cut sites (TCGA). We centered tiles on TaqαI cut sites and screened for uniqueness and complexity using BLASTN (NCBI), comparing tiles to the full genome sequence to reduce cross-reactivity. We excluded tiles with more than one hit greater than 90% sequence similarity. Across the genome, we included 50,935 TaqαI cut sites: 27,128 in protein coding regions, 9,418 within 1,000 bases upstream of genes, and 14,389 in intergenic 'non-coding' regions. The average inter-marker distance was 15.7 kb across the 800 Mb purple urchin genome. We designed control tiles to non-cut sites (TTGA, n = 10,523), ribosomal DNA (positive control for hybridization efficiency, n = 100), and randomly generated tiles that did not match anywhere in the genome according to BLASTN results (negative control for background signal and cross-reactivity, n = 1,036). We also designed a degradation series of tiles in which we randomly changed 1 to 10 bases of a 50-bp tile that matched only one place in the genome (based on BLASTN). We did this for 100 unique tiles, resulting in 1,100 tiles. We used these tiles to estimate the effect of mutational differences between sample DNA and the published genome sequence from which tiles were designed. Tile design was done using MATLAB (2007a, The MathWorks, Natick, MA, USA). All tiles were synthesized in triplicate *in situ *on a 244K-feature high-density custom commercial microarray (Agilent-015554) by Agilent Technologies (Santa Clara, CA, USA). Agilent array probe length is typically 60 bp; 10 'T' nucleotides were first synthesized onto the glass slide before each probe sequence. All raw data files and array platform descriptions have been deposited in NCBI's Gene Expression Omnibus and are accessible through GEO Series accession number [GEO: GSE20857]. Tile names, sequences, and a detailed description of how the characters in the tile name reflect the tile type, position in the genome and gene number are accessible through GEO accession number [GEO: GPL10171].

### DNA processing

We extracted genomic DNA from tube foot tissue using Nucleospin columns following the manufacturer's instructions (Macherey-Nagel, Bethlehem, PA, USA). We randomly sheared 10 μg of DNA per individual, as quantified by NanoDrop (ThermoScientific, Waltham, MA, USA), by sonication (Branson Cell Sonifier, Danbury, CT, USA) for 10 seconds at output control level 3 in a 600 μl volume, followed by ethanol precipitation. Note that although we used 10 μg of DNA as this was readily available in this species, this amount is not required. Based on our experience and Agilent protocols, 250 ng to 1.5 μg are recommended depending on the size of the array used, 60 thousand to 1 million features per array, respectively. We confirmed shearing and DNA recovery on agarose gels (fragment size ranged from 1,000 to 100 bp) and NanoDrop quantification, respectively. We then divided DNA from an individual into two samples of 5 μg each. We treated one sample with a total of 10 units TaqαI restriction enzyme (New England Biolabs, Ipswitch, MA, USA) for 18 hours at 65°C; we then added another 5 units of enzyme for 6 hours. We carried out restriction digestion in 2.5 μg batches in 25 μl reaction volumes using New England Biolabs buffers; we found these conditions important to ensure complete digestion. We heat inactivated the restriction enzyme by incubation at 80°C for 15 minutes. We treated control DNA in the same buffer and temperature conditions, but without the restriction enzyme. We confirmed complete digestion by failure of PCR amplification for an exon with a known TaqαI cut site compared to successful amplification of uncut DNA. We ethanol precipitated DNA before entering labeling reactions. We internally labeled DNA using random octomers and polymerase to incorporate Cy3 (or Cy5) labeled dCTPs (Invitrogen BioPrime labeling and purification kit (Carlsbad, CA, USA), Amersham Cy-dyes (GE Healthcare, Little Chalfont, Buckinghamshire, UK). We labeled non-digested DNA with Cy5-dCTP; we labeled digested DNA with Cy3-dCTP. Labeling efficiency, or specific activity (calculated as picomoles dye per microgram DNA and measured using NanoDrop), was between 80 and 100 pmol dye per microgram DNA for all samples, above the minimum recommended 50 pmol/μg. We carried out ethanol precipitation after sonication and after restriction digestion by adding 1:20 (volume:volume) 3 M sodium acetate and 125 mM EDTA each, then 3:1 (volume:volume) ice cold high-grade 100% ethanol. We quickly vortexed samples then incubated them at -20°C for 15 minutes then spun them at 14,000 g for 30 minutes at 2 to 4°C (TOMY centrifuge, TX-160, Fremont, CA, USA). We found this procedure to yield 95 to 100% DNA recovery based on NanoDrop quantification.

### Microarray processing

We competitively hybridized equal amounts of digested (Cy3-labeled) and non-digested (Cy5-labeled) DNA from an individual to our custom microarray for 40 hours at 65°C, rotating at 20 rpm, following the Agilent protocol for aCGH arrays. Arrays were scanned using a GenePix 4000B scanner (Axon, Molecular Devices, Silicon Valley, CA, USA) set at 5 μm/pixel resolution. We dynamically set PMT gains for 650 (Cy5) and 550 (Cy3) wavelengths for each array such that the overall slide count ratio equaled one. In microarray scanners, the PMT (photomultiplier tube) converts photons into electrical signal, which is then digitized. Note that PMT gains for 650 and 550 wavelengths could be set such that the count ratio equaled one for a subset of tiles on the array, particularly control tiles that are not centered on restriction cut sites (for example, TTGA centered tiles). This would more accurately reflect signal intensity for each channel across the array as equal binding of Cy5 and Cy3 labeled DNA is expected for such control tiles while reduced Cy3 signal intensity is expected for restriction cut site centered tiles (TCGA), the dominant tile type across the array. Though it does not affect the accuracy of polymorphism detection or genotyping, setting the overall slide count ratio equal to one unnecessarily amplifies the Cy3 signal intensity. We extracted and normalized data from the scanned microarray image using Agilent Feature Extraction software. We used the resulting log ratio data (log2 of the ratio of Cy5 (non-digested) signal intensity to Cy3 (digested) signal intensity) to identify polymorphisms and genotype individuals.

### SNP identification

To identify polymorphic loci across the population data set of 20 individuals, we screened for loci with a range in log ratio greater than 0.7 and more than one cluster according to a Bayesian hierarchical clustering algorithm, Mclust [50], implemented in R [75]. We used the average of triplicate tiles for this and subsequent analyses. We used Mclust to determine the number of clusters, from one to four, that best described the log ratio data for all 20 individuals for each locus. We allowed four clusters rather than three as the maximum because the algorithm better assigns three clusters if a fourth is an option [50]. We used a one-dimensional model, parameterization identifier 'VII', with log ratio as input data. Data with one cluster were considered monomorphic. The combined criteria of clusters and log ratio range resulted in trimodal data that reflected the three genotypes of homozygous uncut (low log ratio), heterozygote (intermediate log ratio), and homozygous cut (high log ratio). Note that the homozygous uncut genotype did not result in a log ratio equal to zero (even binding of Cy5 and Cy3) because the whole array image when scanned was normalized for a log ratio equal to zero, offsetting the homozygous uncut genotype to less than zero.

### Sequencing

We designed primers using Primer3 [76] and the published genome sequence [77] to amplify approximately 200 bp within an exon around each restriction cut site. We chose primers such that the 3' end of each primer terminated in the second base position of a codon. We performed PCR amplification using a touchdown protocol for all primer pairs, from 62 to 48°C for 40 cycles. We sequenced amplified DNA using an ABI3100 sequencer.

### Data visualization and analyses

We used MATLAB plotting tools to look at log ratio patterns of the five known polymorphic loci and subsequent loci identified based on Mclust. We used MATLAB functions to perform Kolmogorov-Smirnov tests and correlation statistics. We wrote programs to calculate heterozygosity, F_ST_, and Hardy-Weinberg equilibrium, and to permute the data to simulate panmixia in MATLAB. We used the princomp function in R to perform PCA with loci as rows and samples as columns. We corrected for multiple tests using the Benjamini-Hochberg method [78] and Fisher's combined probability test [79].

## Abbreviations

bp: base pair; EST: expressed sequence tag; GABA: gamma-aminobutyric acid; NCBI: National Center for Biotechnology Information; PCA: Principal Components Analysis; RAD: restriction-site-associated DNA; RSTA: Restriction Site Tiling Analysis; SFP: single feature polymorphism; SNP: single nucleotide polymorphism.

## Authors' contributions

MHP and SRP conceived of the study, MHP developed technical aspects of the method, performed experiments, and performed data analysis with assistance from SRP. TAO designed tile sequences and contributed intellectually to the development of the method. MKM performed gene expression studies. MHP and SRP wrote the manuscript, with contributions from all authors.

## Supplementary Material

Additional file 1 **Table of the top 100 highest F_ST _loci**. The table includes five columns of information: gene number (GLEAN3), gene annotation, RSTA array tile name, RSTA array tile oligonucleotide sequence, and F_ST _value. Click here for file
